# *PDL1* Gene Gain Predicts an Unfavorable Prognosis in HIV-Positive Primary Central Nervous System Lymphoma

**DOI:** 10.3390/curroncol32070378

**Published:** 2025-06-29

**Authors:** Jiamin Chen, Xiaoman Kang, Xinghuan Ding, Yuyang Dai, Lei Sun, Man Li, Ting Liu, Enshan Feng, Xingang Zhou

**Affiliations:** 1 Department of Pathology, Beijing Ditan Hospital, Capital Medical University, Beijing 100015, China; chenjiaminrain@163.com (J.C.); slpumc@126.com (L.S.); liman955190@126.com (M.L.); liuting1981_2005@126.com (T.L.); 2 Department of Neurosurgery, Peking Union Medical College Hospital, Chinese Academy of Medical Sciences and Peking Union Medical College, Beijing 100730, China; xmkang@student.pumc.edu.cn; 3 Department of Neurosurgery, Beijing Ditan Hospital, Capital Medical University, Beijing 100015, China; dingxinghuan0107@163.com; 4 National Institute for Drug Clinical Trial, Beijing Tongren Hospital, Capital Medical University, Beijing 100730, China; daiyy1991@163.com

**Keywords:** primary central nervous system lymphoma, HIV, PDL1, fluorescence in situ hybridization, immunohistochemistry

## Abstract

HIV-positive primary central nervous system lymphoma (PCNSL) shows complex clinical symptoms, progresses rapidly, and carries a poor prognosis. Therefore, effective therapeutic approaches along with novel detection strategies and prognostic markers are urgently needed. Although programmed death ligand 1 (PDL1) is abnormally expressed in a variety of tumors, which correlates with their biological behavior, little is known about its expression and potential role in human immunodeficiency virus (HIV)-positive PCNSL thus far. In this study, we evaluated 41 cases of HIV-positive PCNSL with immunohistochemistry for protein expression and fluorescence in situ hybridization (FISH) for the gene status of *PDL1*. The results showed that PDL1 protein was expressed in 92.7% (38/41) of the cases and in 53.7% (22/41) of the cases with tumor cell proportion score (TPS) > 50%. PDL1 TPS scores were found to be significantly associated with blood CD4+T-cell count (*p* = 0.000), lactate dehydrogenase (LDH) (*p* = 0.020), cerebrospinal fluid (CSF) chloride (*p* = 0.000), location in the lateral ventricles and basal ganglia (*p* = 0.019), activated B-cell (ABC)-like diffuse large B-cell lymphoma (DLBCL) (*p* = 0.004), necrosis area (*p* = 0.000), CD10 (*p* = 0.000), BCL6 (*p* = 0.003), BCL2 (*p* = 0.005), c-MYC (*p* = 0.003), Epstein–Barr encoding region (EBER) (*p* = 0.000), and PD1 TPS (*p* = 0.039). A total of 46.3% (19/41) of cases showed *PDL1* gene gain. The gain of *PDL1* status was positively correlated with blood CD4+T-cell count (*p* = 0.028), necrosis area (*p* = 0.011), and BCL6 (*p* = 0.050). It was discovered that the *PDL1* gene gain was correlated with protein overexpression (*p* = 0.004). Survival analyses showed that *PDL1* gain was significantly associated with worse survival (*p* = 0.024). Multivariate analyses demonstrated that *PDL1* gain was an independent predictive factor for poor prognosis (*p* = 0.043). Despite the limited cohort size, these findings suggest that *PDL1* gain could be considered a potential biomarker for prognosis in the context of PD1/PDL1 therapy.

## Simple Summary

In HIV-positive patients, primary central nervous system lymphoma (PCNSL) is a dangerous brain tumor. Our study of 41 cases revealed that nearly all tumors (93%) exhibit elevated PDL1 expression—an immune checkpoint protein often dysregulated in cancers. Crucially, 46% of tumors harbored increased *PDL1* gene copy numbers, directly associated with more aggressive tumor behavior. Patients with these genetic alterations had significantly shorter survival times, establishing *PDL1* copy number gain as a potential prognostic marker for poor outcomes. Detecting these *PD-L1* gene amplifications helps clinicians identify the highest-risk patients. Importantly, these findings strongly suggest that existing PD1/PDL1 immune checkpoint inhibitors may represent a promising targeted therapy for this vulnerable patient group, offering potential for improved future treatments.

## 1. Introduction

Primary central nervous system lymphoma (PCNSL) refers to non-Hodgkin lymphoma (NHL) originating in the brain, eyes, spinal cord, and cerebrospinal fluid without the presence of lymphoma outside of the central nervous system [1]. In the 2022 edition of the WHO Classification of Haematolymphoid Tumours, PCNSL is classified within the group of “large B cell lymphomas of immune-privileged sites.” The incidence of the disease in immune abnormalities (acquired immune deficiency syndrome (AIDS), organ transplantation, or immunosuppressive agent application) is significantly higher than that in the normal population, suggesting that the disease is associated with immunodeficiency [2]. PCNSL has slow onset, diffuse growth, and high malignancy, and relapses easily, rendering treatment extremely challenging.

Viral infections are increasingly recognized as drivers of oncogenesis, with mechanisms involving both direct viral oncoprotein activity and immune modulation. The Epstein–Barr virus (EBV), classified by the International Agency for Research on Cancer (IARC) as a Group 1 carcinogen, promotes tumor development through latent proteins that disrupt cellular growth controls, particularly in immunosuppressed hosts [3]. Notably, HIV enhances cancer risk through dual pathways: chronic inflammation from persistent immune activation and CD4+ T-cell depletion-induced immunosuppression, which collectively increase susceptibility to co-infections with oncogenic viruses like EBV [4]. EBV-positive and EBV-negative PCNSL exhibit distinct molecular profiles, supporting their classification as biologically distinct subtypes [5,6,7].

Our previous study also showed that HIV-positive PCNSL showed unique clinical pathological significance and molecular features [8]. The programmed death ligand 1(*PDL1*) gene located at chromosome 9p24.1, which encodes PDL1, a negative regulatory signal mediating the immune response, plays specific regulatory roles in tumorigenesis, viral infection, and autoimmune diseases [9]. PDL1 overexpression is widely observed in malignancies and correlates with disease progression, serving as both a therapeutic target and a prognostic indicator. Oncoviruses evade immune surveillance through multiple strategies, including suppressing antigen presentation, elevating PDL1 expression, and activating immunosuppressive cells, which collectively hinder antitumor immunity [5,6,10,11,12]. The aim of this study was to evaluate the gene and protein status of PDL1 in HIV-positive PCNSL, as well as to explore the potential prognostic value of them in these lesions.

## 2. Materials and Methods

### 2.1. Patients and Tumour Samples

**Figure 1:**
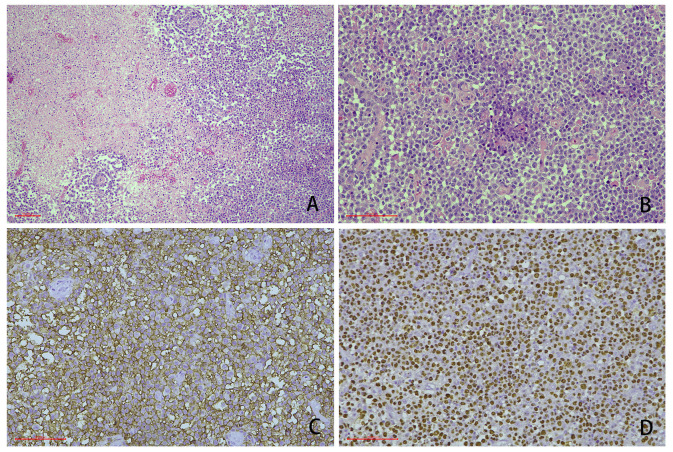
Representative cases showing the typical morphology of human immunodeficiency virus (HIV)-positive primary central nervous system lymphoma (PCNSL) (scale bar: 100 μm). (**A**) The cases were characterized by a diffuse infiltration of medium to large cells with a large necrotic area. In some cases, the tumor cells were prone to growing around blood vessels (HE staining, 100-fold magnification). (**B**) The tumor cells had large nucleoli and abundant cytoplasm, and some tumor cells were accompanied by plasmacytoid features (HE staining 200-fold magnification). (**C**) CD20 immunohistochemical staining: the neoplastic cells expressed pan B-cell markers (400-fold magnification). (**D**) Epstein–Barr encoding region (EBER) in situ hybridization: most of the cells were positive (400-fold magnification).

Our retrospective study analyzed 41 formalin-fixed, paraffin-embedded (FFPE) tissues from treatment-naïve HIV-positive patients (31 surgical resections, 10 biopsies) collected at Beijing Ditan Hospital, Capital Medical University, between January 2008 and February 2022. All cases were histopathologically reclassified according to the World Health Organization Classification of Haematolymphoid Tumours (5th edition), identifying 39 primary central nervous system lymphomas (PCNSLs; 95.1%) and 2 Burkitt lymphomas (4.9%). Immunohistochemical analysis of PCNSL subtypes revealed 32 cases (82.1%) with an activated B-cell-like (ABC) phenotype and 7 cases (17.9%) with a germinal center B-cell-like (GCB) immunoprofile (Figure 1) [13]. Among the 41 cases, 30 cases (73.2%) were positive for Epstein–Barr encoding region (EBER) in situ hybridization and 11 cases (26.8%) were negative for EBER in situ hybridization (Supplementary Table S1). The study protocol, approved by the Institutional Ethics Committee of Beijing Ditan Hospital (DTEC-KY2024-053-01), adhered to the Helsinki Declaration. Preoperative evaluations encompassed immune status (CD4+ T-cell counts, HIV/EBV viral loads), metabolic profiling (serum lactate dehydrogenase), cerebrospinal fluid (CSF) analysis (protein, glucose, chloride levels), and neuroimaging-guided lesion localization (MRI and intraoperative confirmed). Necrotic regions were histologically validated by board-certified pathologists. All data were derived from baseline assessments prior to therapeutic interventions.

### 2.2. Immunohistochemistry (IHC) and In Situ Hybridization

Immunohistochemical testing included PDL1, PD1, CD10, BCL6, MUM1, BCL2, c-MYC, P53, and Ki-67. Epstein–Barr encoding region (EBER) testing was performed by in situ hybridization. All IHC was detected using the EnVision method. The immunohistochemical staining for PDL1 and PD1 was performed in strict accordance with the manufacturer’s protocols and carried out manually. The primary monoclonal antibodies were rabbit anti-human PDL1 (monoclonal, clone E1L3N; Maijie Translational Medicine Research, Suzhou, Co., Ltd., Suzhou, China) and mouse anti-human PD1 (monoclonal, clone UMAB199; Beijing Zhongshan Jinqiao Biotechnology Co., Ltd., Beijing, China). The second antibody reagents were purchased from Beijing Zhongshan Jinqiao Biotechnology Co., Ltd., Beijing, China. All reactions were performed with appropriate positive and negative controls. The reactive hyperplasia of the amygdala was used as a positive control, and tissues with PBS instead of the primary antibody were used as a negative control. The PDL1 protein is localized to the cell membrane, while the PD1 protein is localized to both the cell membrane and the cytoplasm.

Other immunohistochemical testing included CD10 (clone UMAB235), BCL6 (clone LN22), MUM1 (clone EP190), BCL2 (clone OT1R1H2), c-MYC (clone EP121), P53 (clone DO-7), and Ki-67 (clone UMAB107). The primary antibodies for immunohistochemical analysis were procured from Zhongshan Jinqiao Biotechnology (Beijing, China). The EBER in situ hybridization detection system (BOND Ready-to-Use format) was acquired from Leica Biosystems (Shanghai, China). All experimental procedures, including immunohistochemical staining and in situ hybridization, were conducted using a LEICA BOND-MAX automated staining platform. Appropriate positive and negative control samples were systematically incorporated throughout the experimental workflow to ensure assay validity.

All valid tumor cells were evaluated on the whole section, and at least 100 tumor cells were counted. The tumor cell proportion score (TPS), defined as the percentage of tumor cells exhibiting membranous PDL1 staining (regardless of intensity), was calculated independently. The positive fraction was the combined positive score (CPS), or the sum of tumor cells with PDL1 staining and tumor-associated immunity (including lymphocytes and macrophages) per 100 tumor cells. Only the proportion of cell membrane staining of tumor cells was calculated, without considering the staining intensity and staining integrity of the cell membrane. Immunostaining was assessed semiquantitatively based on the percentage of positive cells in the tissues. Two pathologists (XGZ and JMC) independently scored cases, with a concordance rate of 100%. The PDL1 TPS scoring criteria were as follows: the section was scored as 0 for TPS = 0, 1 for TPS = 1–20%, 2 for TPS = 21–50%, and 3 for TPS > 50% tumor cells expressed. The scoring criteria for PDL1 CPS were as follows: a scored of 0 for CPS = 0–19, 1 for CPS = 20–49, 2 for CPS = 50–100, and 3 for CPS > 100. The scoring criteria for PD1 also follow the aforementioned standards.

The other immunohistochemical scoring criteria were defined as follows. P53: Scores were assigned based on the percentage of tumor cells with nuclear staining: 1 (5–25%), 2 (26–50%), and 3 (>50%). Ki-67: The proliferation index was categorized as 1 (<20% nuclear staining), 2 (21–50%), or 3 (>50%). c-MYC: Expression levels were classified as low (<40% nuclear staining) or overexpression (≥40%). BCL2: Cytoplasmic staining intensity was categorized as low (<50% positive tumor cells) or overexpression (≥50%). BCL6/MUM1 and CD10: Positivity thresholds were defined as >30% of tumor cells exhibiting distinct nuclear (BCL6/MUM1) or membranous (CD10) staining.

*PDL1* gene amplification was evaluated by FISH with a *PD-L1*(9p24)/CSP9 probe kit (Guangzhou LBP Medical Technology, China). Briefly, 4 μm-thick tissue sections were deparaffinized and hydrated, followed by antigen retrieval in purified water at 95–100 °C for 14 min. Slides were then digested with pepsin working solution (37 °C, 12 min), dehydrated, and hybridized with probes using a StatSpin^®^ Abbott ThermoBrite A automated hybridization system. Co-denaturation (75 °C, 20 min) and hybridization (37 °C, 24 h) were performed sequentially. Post-hybridization washes were conducted in 2 × SSC/0.3% NP-40 buffer, and nuclei were counterstained with 4′,6-diamidino-2-phenylindole (DAPI). Fluorescent signals were independently analyzed by two blinded investigators using a Nikon 80i fluorescence microscope (Nikon, Tokyo, Japan). A minimum of 20 interpretable interphase nuclei were scored per specimen. *PDL1* status was determined according to previous studies. *PDL1* gene amplification was described as a *PDL1*/centromere of chromosome 9 (PDL1/CEP9) ratio > 2.0, while *PDL1* deletion was described as a PDL1/CEP9 ratio ≤ 0.8. The case was regarded as gain when the ratio of *PDL1*/CEP9 was between 1.2 and 2.0 [14]. Both red and green signals with >2.0 were polyploidy. Images of nuclei were captured by NIS-Elements BR 3.0 (NIKON, Tokyo, Japan).

### 2.3. Statistical Analysis

Statistical analyses were performed using SPSS 23.0 (IBM Corp., Armonk, NY, USA). Continuous variables are presented as mean ± SD and analyzed using Student’s *t*-test (two-group comparisons) or one-way ANOVA (multi-group comparisons). Categorical variables were evaluated with the χ² test or Fisher’s exact test. Survival outcomes were assessed via Kaplan–Meier curves with log-rank testing (univariate) and Cox proportional hazards regression (multivariate). Statistical significance was defined as *p* < 0.05.

## 3. Results

### 3.1. PDL1 Protein Expression

**Figure 2:**
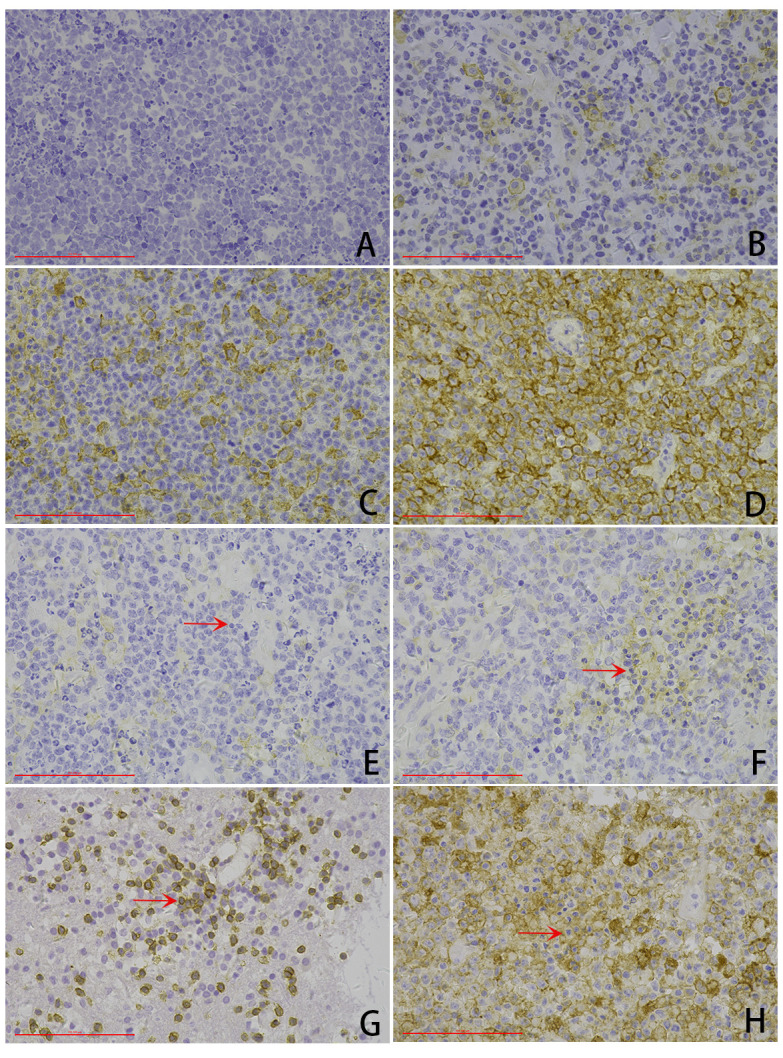
Representative microphotography of PDL1 immunohistochemical expression (scale bar: 100 μm). (**A**–**D**) Tumor cell proportion score (TPS): (**A**) TPS score of 0, (**B**) TPS score of 1, (**C**) TPS score of 2, (**D**) TPS score of 3. (**E**–**H**) Combined positive score (CPS): (**E**) CPS score of 0, (**F**) TPS score of 1, (**G**) CPS score of 2, (**H**) CPS score of 3. Arrows: tumor-associated immunity.

Of the 41 cases, 7.3% (3/41) of the cases had a TPS score of “0,” 22.0% (9/41) of the cases had a TPS score of “1,” 17.1% (7/41) of the cases had a TPS score of “2,” and 53.7% (22/41) of the cases had a TPS score of “3,” (Figure 2). PDL1 TPS scores were found to be significantly positively correlated with lower blood CD4+T-cell count (*p* = 0.000), lower LDH (*p* = 0.020), higher CSF chloride (*p* = 0.000), location in the lateral ventricles and basal ganglia (*p* = 0.019), ABC-like DLBCL (*p* = 0.004), necrosis area (*p* = 0.000), higher expression of BCL2 (*p* = 0.005), lower expression of c-MYC (*p* = 0.003), positive expression of EBER (*p* = 0.000), and lower PD1 TPS (*p* = 0.039).

PDL1 TPS scores were not associated with other clinicopathological characteristics such as blood EBV nucleic acid quantification (*p* = 0.101), blood HIV viral load (*p* = 0.742), CSF protein (*p* = 0.635), CSF sugar (*p* = 0.080), gender (*p* = 0.552), age (*p* = 0.062), P53 (*p* = 0.295), Ki-67 (*p* = 0.057), and PD1 CPS (*p* = 0.058) (Supplementary Table S1).

Of the 41 cases, 19.5% (8/41) of the cases had a CPS score of “0,” 12.2% (5/41) of the cases had a CPS score of “1,” 19.5% (8/41) of the cases had a CPS score of “2,” and 48.8% (20/41) of the cases had a CPS score of “3” (Figure 2). PDL1 CPS scores were found to be significantly positively correlated with lower blood CD4+T-cell count (*p* = 0.000), lower CSF sugar (*p* = 0.040), ABC-like DLBCL (*p* = 0.007), necrosis area (*p* = 0.000), higher expression of Ki-67 (*p* = 0.033), positive expression of EBER (*p* = 0.000), and lower PD1 CPS (*p* = 0.021).

PDL1 CPS scores were not associated with other clinicopathological characteristics, such as site (*p* = 0.059), blood EBV nucleic acid quantification (*p* = 0.101), LDH (*p* = 0.676), blood HIV viral load (*p* = 0.157), CSF protein (*p* = 0.535), CSF chloride (*p* = 0.225), gender (*p* = 0.843), age (*p* = 0.195), P53 (*p* = 0.151), BCL2 (*p* = 0.135), and PD1 TPS (*p* = 0.137) (Supplementary Table S1).

### 3.2. PDL1 Copy Number Alteration

**Figure 3:**
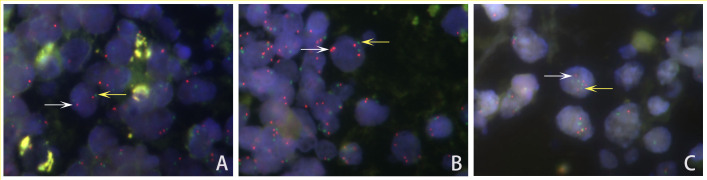
Representative FISH image of HIV-positive PCNSL cells showing *PDL1* gene status. (**A**) Normal: Cells with 2 *PDL1* signals (red, white arrow) and 2 CEP9 signals (green, yellow arrow). (**B**) Gain: The ratios of *PDL1* signals (red, white arrow)/CEP9 signals (green, yellow arrow) in the tumor cell nuclei were between 1.2 and 2.0. (**C**) Polyploidy: Both numbers of *PDL1* signals (red, white arrow) and CEP9 probe signals (green, yellow arrow) equaled more than 2.0 copies.

Of the 41 cases, 46.3% (19/41) of the cases showed *PDL1* gene gain, and 9.8% (4/41) of the cases exhibited polyploidy of chromosome 9 (Figure 3). The gain of *PDL1* status was positively correlated with lower blood CD4+T-cell count (*p* = 0.028) and necrosis area (*p* = 0.011).

*PDL1* gain had no correlation with LDH (*p* = 0.566), blood EBV nucleic acid quantification (*p* = 0.154), blood HIV viral load (*p* = 0.136), CSF sugar (*p* = 0.629), CSF protein (*p* = 0.678), CSF protein (*p* = 0.678), CSF chloride (*p* = 0.430), gender (*p* = 0.257), age (*p* = 0.491), site (*p* = 0.816), type (*p* = 0.098), P53 (*p* = 0.663), Ki-67 (*p* = 0.519), BCL2 (*p* = 0.193), c-MYC (*p* = 0.131), EBER (*p* = 0.173), PD1 TPS (*p* = 0.447), or PD1 CPS (*p* = 0.615) (Supplementary Table S2).

*PDL1* gain was associated with PDL1 TPS, and high expression of PDL1 in tumor cells was more likely to occur in cases of *PDL1* gain (*p* = 0.004). However, it was not associated with PDL1 CPS (*p* = 0.062) (Table 1).

### 3.3. Survival Analysis

A total of 35 cases were included in follow-up analyses. Three cases were excluded with survival time ≤ 15 days, considering the death related to post-complications rather than the disease itself. The remaining patients underwent high-dose methotrexate-based chemotherapy. In all 32 patients, 18 were alive and 14 dead, and overall survival (OS) ranged from 18 to 1948 days, with average OS of 438.7 days and a median OS of 213.5 days. In 18 cases with *PDL1* gain, 7 patients were alive (38.89%, 7/18) and 11 dead (61.11%, 11/18), and OS ranged from 18 to 1394 days, with a median survival time of 109.5 days. By contrast, in 14 cases without *PDL1* gain, 11 patients survived (78.57%, 11/14) and 3 died (21.43%, 3/14), and OS ranged from 23 to 1948 days, with a median survival time of 705 days.

**Figure 4:**
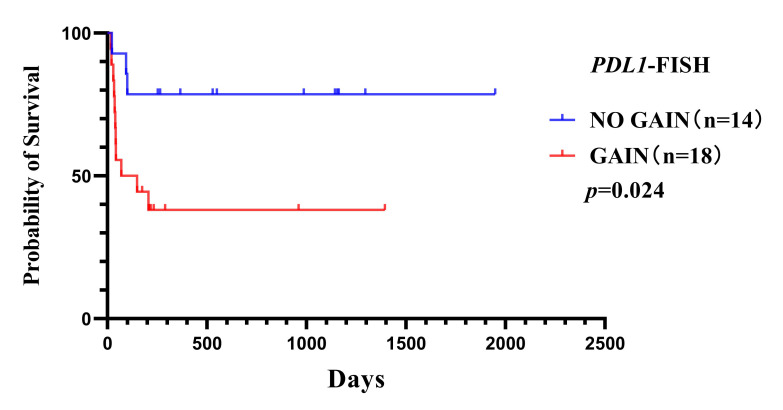
Overall survival curves for HIV-positive PCNSL patients with or without *PDL1* gene gain (*p* = 0.024).

Kaplan–Meier analysis and a log-rank test showed a statistically significant difference in survival rate between patients with and without *PDL1* gain (log-rank test *p* = 0.024). Tumors with *PDL1* gain were associated with worse survival (Figure 4). Then, Cox regression verified the independence of the clinical prognostic significance of *PDL1* gain in HIV-positive PCNSL after adjusting CSF sugar in the HIV-positive PCNSL cohort (*p* = 0.043) (Table 2).

## 4. Discussion

PCNSL has been regarded as presenting AIDS-related tumors and shows complex clinical symptoms, progresses rapidly, and carries a poor prognosis. With anti-retrovirus (ART) therapy widely applied in clinical treatment, AIDS has gradually become a chronic and nonfatal disease [15]. With the prolonged survival time of AIDS patients, malignancy has gradually emerged as a pivotal factor threatening HIV/AIDS survival and the well-being of these patients. Finding new treatment strategies is particularly important for prolonging the survival of patients with PCNSL, especially those with HIV infection. Both PD1 and the ligands of PD1 (PDL) are B7 family molecules. By overcoming the suppression of the immune system induced by tumor cells, the therapeutic approach of PD1/PDL1 immune checkpoint inhibition improves the possibility of patients’ organisms being able to fight cancer [16].

Regarding the research on PDL1, the findings differ between solid tumors and lymphoma (Supplementary Table S3). It has been proven that the PDL1 protein is highly expressed in solid cancers, such as non-small-cell lung cancer, gastric cancer, adrenocortical carcinoma (ACC), small-cell carcinoma of the esophagus, and inflammatory myofibroblastic tumor [17,18,19,20,21].

The study by Berghoff et al. showed that 10% of patients with PCNSL were positive for PDL1 [22]. Georgiou et al. proved that 26.4% to 75% of the assessed tumors were positive for PDL1 expression in the case of DLBCL. PDL1 expression was more frequently observed in non-GCB cases [23].

PDL1 overexpression also appeared in classical Hodgkin’s lymphoma (cHL), primary mediastinal B-cell lymphoma (PMBCL), and nodular lymphocyte predominant Hodgkin lymphoma (NLPHL) [24]. In other B-cell lymphoma entities (5% in follicular lymphoma; 10% in high-grade marginal zone lymphomas, and none in mantle-cell lymphoma), PDL1 is only expressed in a low percentage of cases [25,26].

In T-cell lymphomas, PDL1 expression is observable in peripheral T-cell lymphoma (PTCL), anaplastic large-cell lymphoma (ALCL), angioimmunoblastic T-cell lymphoma (AITL), extranodal NK/T-cell lymphoma of the nasal type, cutaneous T-cell lymphoma, and adult T-cell leukemia/lymphoma [27].

Our results showed that over 90% of cases with PCNSL were positive for PDL1 protein expression in tumor cells, and 50% of these cases had high PDL1 expression, higher than the values reported in the literature. This may be due to the high infection rate of EBV in our cases. As a carcinogen, EBV promotes tumor development by expressing latent proteins that disrupt cellular growth controls, particularly in immunosuppressed hosts. In the case of AIDS, the disease involves a state of chronic inflammation and immunosuppression caused by infected cells, making individuals more susceptible to infection with several oncogenic viruses and complicating the clearance of these viruses [3,4]. It has been shown that PDL1 protein expression can be induced by the latent membrane protein 1 (LMP1) of EBV via activation of STAT (particularly STAT3)- and activated protein1 (AP1)-mediated pathways, which was observable in cHL and DLBCL [28]. Due to the critical molecular differences, EBV-associated (EBV+) and EBV-negative (EBV-) PCNSL were considered distinctive subtypes [5,6,7]. All EBV-associated malignancies were anticipated to be PDL1 positive in PCNSL [7], peripheral T-cell lymphoma, ALK-positive ALCL, extranodal NK/T-cell lymphoma of the nasal type, cutaneous T-cell lymphoma, and adult T-cell leukemia/lymphoma [27]. Our analysis further demonstrated a significant association between PDL1 positivity and EBER expression, reinforcing the critical role of immune evasion mechanisms in CNS lymphoma development within immunocompromised populations.

Research on *PDL1* gene copy number variation shows that *PDL1* amplification is rare in ACC [19] and cervical cancer [29]. *PDL1* gain and/or amplification was detected in squamous cell cancer of the lung (4.5% amplification) [30], non-small-cell lung cancer (3.1% amplification) [31], advanced melanomas (13.8% amplifications and 22.2% gains) [14], and squamous-cell carcinoma of the oral cavity [32] (19% amplification). Goldmann et al. showed that no association was seen between the *PDL1* amplification and other clinicopathological parameters in squamous-cell cancer of the lung [30]. However, Inoue et al. proved that *PDL1* copy number gains were associated with smoking-related tumors, and *PDL1* gene amplification was independently associated with high immune infiltrates, EGFR expression, and regional lymph node metastases in non-small-cell lung cancer [31].

Alterations in 9p24.1 have been detected in cHL [33], mainly the nodular sclerosis subtype and some specific subsets of DLBCL such as PMBCL, primary testicular lymphoma (PTL), primary central nervous system DLBCL, and PMBCL [23,34,35]. Our research revealed that among the 41 cases examined, 46.3% exhibited gains in the *PDL1* gene, and 9.8% showed polyploidy of chromosome 9. These findings were consistent with recent studies on PMBCL, PCNSL, PTL, and others [10]. Georgiou et al. found gains (6–19%) and amplifications (3–3.5%) of the *PDL1* locus in DLBCL [23]. Furthermore, they proved that *PDL1* loci were more frequently observed in a younger age and the non-GCB subtype of DLBCL. They also found that the amplification frequency was 29% in PMBCL. Differently, we did not find *PDL1* amplification in our research. Our study showed that *PDL1* gains were more likely to occur in cases with higher blood CD4+T-cell count, a larger necrosis area, and BCL6 negativity, but were not related to other clinical pathological indicators, including histological types.

These research findings above are not consistent in T-cell lymphoma. Manso et al. [27] showed that there were no amplifications, deletions, or rearrangements of *PDL1* in peripheral T-cell lymphomas. In contrast, in a recent study by Gerbe et al. [36] found that in 21.1% and 8.3% of systemic and cutaneous ALK-negative ALCL, respectively, *PDL1* amplification occurs.

The relationship between *PDL1* gene amplification and PDL1 expression was also inconsistent in some solid cancers and different types of lymphomas. *PDL1* gene amplification was associated with PDL1 expression in non-small-cell lung cancer [17], the oral cavity [32], and squamous-cell cancer of the lung [28]. There was no significant relationship between PDL1 expression and *PDL1* copy number variations in cervical cancer [29] and advanced melanomas [14].

Our research confirmed that high expression of PDL1 was associated with *PDL1* gain. This finding is consistent with studies conducted on cHL, PMBCL, and PCNSL. However, according to the study by Manso et al., there was no correlation between PDL1 expression in tumor cells and 9p24.1 gene region alterations in peripheral T-cell lymphomas [27]. These findings suggest that PDL1 expression can be regulated through multiple mechanisms beyond direct genetic aberrations. Notably, emerging evidence has demonstrated that JAK/STAT pathway activation serves as an alternative regulatory mechanism for PDL1 induction [37]. Actual protein expression can be affected by post-transcriptional regulation, such as microRNA suppression and protein degradation, or by dynamic factors within the tumor microenvironment, including inflammatory cytokines. The expression of the PDL1 protein may undergo dynamic adjustments due to therapeutic pressures, such as immunotherapy or changes in the tumor microenvironment, which could result in inconsistent prognostic implications between the two.

PDL1 expression was related to a poor prognosis in patients with gastric cancer [38]. In contrast, it indicated a favorable prognosis in ACC [19] and small-cell carcinoma of the esophagus [20]. PDL1 overexpression tended to correlate with better responses to chemotherapy in breast carcinomas and thymic epithelial neoplasms [39]. Survival analysis did not reveal any association with either the *PDL1* amplification in squamous-cell cancer of the lung [30] or thymic epithelial neoplasms [39]. In non-small-cell lung cancer, Inoue et al. proved that both *PDL1* amplification and the level of protein expression were predictors of poor survival [31], while Koh et al. did not find any association between PDL1 expression and OS or relapse-free survival (RFS) rate [17].

Our research indicated that *PDL1* gain was an independent unfavorable prognostic factor in HIV-positive PCNSL, consistent with the findings of Xu-Monette et al. in cHL [26], and Twa and Camus et al. in PMBCL [34,35]. Kiyasu and Cheng et al. also showed that PDL1 overexpression in DLBCL was validated to be an independent predictor of poor prognosis, especially in ABC-DLBCL [24,40]. Chapuy et al. proved that PDL1 protein expression was associated with poorer OS but was not an independent predictor of OS in PTL and PCNSL [10]. On the contrary, Wang et al. showed that the cases with a 9p24.1 amplification had a trend of better event-free survival in DLBCL [41]. Ramsay and Gamaleldin et al. indicated that PDL1 expression was associated with poor prognosis in chronic lymphocytic leukemia (CLL)/small lymphocytic lymphoma (SLL), and was associated with failure of complete remission, shorter progression-free survival, and shorter overall survival in CLL [28], but Menter and Zhang et al. showed that PDL1 expression in CLL/SLL had no prognostic significance in most studies [25,42].

Similar to our research findings, Loharamtaweethong et al. also found *PDL1* amplification, polyploidy of chromosome 9, and PDL1 protein overexpression in HIV-positive cervical cancer, which may also serve as a biomarker for prognosis of PD1/PDL1 therapy [42]. For HIV-positive patients, PDL1 inhibitors help to restore the effector function and proliferation capacity of specific CD8+ T cells, and have a dual role in tumor immunotherapy [43]. Research by Bari et al. indicates that PDL1 inhibitors were safe and effective for HIV-positive tumor patients [44].

This study has several limitations that should be acknowledged. Firstly, the relatively small sample size may diminish the statistical power of our analyses and limit the generalizability of the findings. Secondly, the investigative approach was limited to immunohistochemical and FISH assays, potentially overlooking underlying molecular mechanisms that could be elucidated through more advanced techniques. Finally, the absence of in-depth mechanistic investigations prevents definitive conclusions regarding causal relationships. Future research that includes larger multicenter cohorts, integrated multi-omics approaches, and experimental validation would help to address these constraints.

In the present study, we initially studied the situation of *PDL1* gene and protein expression in HIV-positive PCNSL. *PDL1* gene gain was correlated with protein overexpression and worse survival. Multivariate analyses demonstrated that *PDL1* gain was an independent predictive factor for poor prognosis, offering new insights and more possibility into its prognosis significance and PDL1 inhibitor treatment.

## Figures and Tables

**Table 1:** Association between *PDL1* gene and PDL1 expression in HIV-positive PCNSL.

		PDL1 Protein Expression									
		TPS					CPS				
		0	1–20%	21–50%	>50%	*p* Value	0–19	20–49	50–100	>100	*p*-Value
*PDL1* gene	Gain	0	2	2	18	*p* = 0.004 *	1	2	4	15	*p* = 0.062
	Nogain	1	7	5	4		5	3	4	5	

Note. TPS = tumor cell proportion score, CPS = combined positive score. Chi-square test and rank correlation were used. * *p* < 0.05.

**Table 2:** Univariate and multivariate Cox regression analysis in the HIV-positive PCNSL cohort.

Characteristic	Univariate Analysis			Multivariate Analysis	
	HR (95% CI)	*p* Value		HR (95% CI)	*p* Value
PDL1 FISH	3.942 (1.094–14.209)	0.036		3.820 (1.043–13.983)	0.043
CSF sugar	0.301 (0.102–0.884)	0.029		—	—

Note. The multivariable Cox proportional hazards regression model was constructed using the forward conditional method for variable selection.

## Data Availability

Data relating to this study can be made available by contacting the corresponding authors.

## Supplementary Materials

The following supporting information can be downloaded at: https://www.mdpi.com/article/10.3390/curroncol32070378/s1, Table S1: Association between PDL1 expression and clinico-pathological features in HIV-positive PCNSL; Table S2: Association between *PDL1* gene and clinico-pathological features in HIV-positive PCNSL; Table S3: PDL1 protein and gene in tumors.
