# Supplementary material for: PDL1 Gene Gain Predicts an Unfavorable Prognosis in HIV-Positive Primary Central Nervous System Lymphoma
Source: Curr Oncol. 2025 Jun 29;32(7):378. doi: 10.3390/curroncol32070378 (PMC12294013; doi:10.3390/curroncol32070378)
Supplement: Supplementary file 1 [file curroncol-32-00378-s001.zip › curroncol-3606393-supplementary.pdf]

Supplementary Table S1. Association between PDL1 expression and clinico-pathological features in HIV-positive PCNSL

		PDL1 protein expression									
		TPS					CPS				
		0	1-20%	21-50%	>50%	*P* value	0-19	20-49	50-100	＞100	*P* value
**Type** PCNSL	GCB	0	6	0	1	*P* = 0.004*	4	1	1	1	*P* = 0.007*
	ABC	1	3	7	21		2	4	7	19	
	BL	2	0	0	0	-	2	0	0	0	-
**Male/Female**		3/0	7/2	5/2	20/2	*P = 0.552*	7/1	5/0	5/3	18/2	*P* = 0.843
**Age (years)**	< 45	1	5	4	18	*P = 0.062*	3	4	6	15	*P* = 0.195
	≥ 45	2	4	3	4		5	1	2	5	
**Site**	lateral ventricle&basal ganglia	0	5	5	14	*P = 0.019**	3	2	6	13	*P* = 0.059
	Supra& infratentorial	1	3	2	8		3	2	2	7	
	Spinal canal	2	1	0	0		2	1	0	0	
**Blood CD4+T cell count (cells/μl)**		671.67±390.08	385.89±232.62	134.14±279.53	33.50±35.04	*P = 0.000***	572.50±274.11	132.60±174.97	135.75±269.85	41.75±36.78	*P* = 0.000**

**Blood HIV viral load(copies/ml)**		13290.33±11528.42	243374.22±69759.43	207520.86±226428.72	121066.71±200793.77	*P* = 0.742	15099.00±19134.73	479092.40±917023.13	129547.13±222779.48	140666.05±208287.24	*P* = 0.157
**Blood EBV nucleic acid (copies/ml)**		26823.00±36737.03	204230.00±342053.70	57503.33±91409.92	20145.21±35072.37	*P* = 0.101	23191.50±25843.86	308600.00±355441.53	50505.00±75425.44	12073.47±17377.09	*P* = 0.101
**LDH (U/L)**		639.47±634.09	222.96±77.54	216.00±44.67	234.89±151.79	*P = 0.020**	373.49±444.85	297.78±46.11	241.61±61.36	337.44±163.55	*P* = 0.676
**CSF protein (mg/dl)**		194.50±13.01	104.50±95.84	103.88±94.30	126.88±93.08	*P* = 0.635	149.12±95.06	94.30±61.53	76.44±73.73	131.33±96.92	*P* = 0.535
**CSF sugar (mmol/l)**		2.83±0.36	3.98±1.50	2.66±1.37	2.78±0.76	*P* = 0.080	4.12±1.34	3.26±1.26	2.96±0.93	2.64±0.89	*P* = 0.040*
**CSF chloride (mmol/l)**		59.60±84.29	120.10±5.35	120.98±8.29	122.84±7.19	*P = 0.000***	100.97±49.56	117.80±7.16	124.80±3.41	121.82±7.98	*P* = 0.225
**necrotic areas**	≤ 25%	3	8	4	7	*P = 0.000***	8	3	6	5	*P = 0.003**
	26-50%	0	0	1	4		0	0	0	5	
	≥ 50%	0	1	2	11		0	2	2	10	
**CD10**	> 30%	2	6	0	1	*P = 0.000***	2	4	7	19	*P = 0.001***
	≤ 30%	1	3	7	21		6	1	1	1	

**BCL-6**	Positive	1	6	0	1	*P = 0.003**	5	2	1	0	*P = 0.000***
	negtive	2	3	7	21		3	3	7	20	
**MUM-1**	Positive	3	6	7	22	*P = 0.051*	7	3	8	20	*P = 0.067*
	negtive	0	3	0	0		1	2	0	0	
**BCL-2**	≥ 50%	0	4	5	17	*P = 0.000***	2	3	8	13	*P = 0.135*
	＜ 50%	3	5	2	5		6	2	0	7	
**C-MYC**	≥ 40%	2	6	4	2	*P = 0.003**	6	3	2	3	*P = 0.003**
	＜ 40%	1	3	3	20		2	2	6	17	
**P53**	≤ 25%	0	4	1	1	*P = 0.295*	3	1	1	1	*P = 0.151*
	26-50%	0	1	2	5		0	2	2	4	
	＞ 50%	3	4	4	16		5	2	5	15	
**Ki67**	21-50%	0	0	0	5	*P = 0.057*	0	0	0	5	*P = 0.033**
	＞ 50%	3	9	7	17		8	5	8	15	
**DELs**	yes	0	2	3	2	*P = 0.304*	2	1	2	2	*P = 0.059*
	no	1	7	4	20		4	4	6	18	
**EBER**	positive	0	3	6	21	*P = 0.000***	2	3	6	19	*P = 0.000***
	negtive	3	6	1	1		6	2	2	1	

**PD1**	TPS = 0	3	5	4	10		7	2	3	10	
	TPS = 1-20%	0	3	2	5	*P* = 0.039*	0	2	4	4	*P = 0.137*
	TPS = 21-50%	0	0	0	4		0	1	0	3	
	TPS>50%	0	1	1	3		1	0	1	3	
	CPS = 0-19	3	5	1	6		7	2	1	5	
	CPS = 20-49	0	1	3	7	*P* = 0.058	0	0	5	6	*P = 0.021**
	CPS = 50-100	0	3	3	6		1	3	2	6	
	CPS > 100	0	0	0	3		0	0	0	3	

NOTE. Continuous variables are presented as mean ± SD or median. GCB = germinal centre B-cell like diffuse large B-cell lymphoma, ABC = activated B-cell like diffuse large B-cell lymphoma, BL = Burkitt lymphoma, HIV = human immunodeficiency virus, EBV = Epstein-Barr virus, LDH = dehydrogenase, CSF = cerebrospinal fluid, TPS = tumor cell proportion Score, CPS = combined positive score, DEL = double expressor lymphoma, EBER = Epstein-Barr encoding region.

Student's t-test，chi-square test and rank correlation were used.

* $p < 0.05$.

** $p < 0.01$.

Supplementary Table S2. Association between *PDL1* gene and clinico-pathological features in HIV-positive PCNSL

			***PDL1* gene**			***P* value**
			**Gain**	**No gain**	**Gain Rate**	
**Type**	PCNSL	GCB	1	6	14.29%（1/7）	*P* = 0.098
		ABC	17	15	53.13%（17/32）	
	BL		1	1	50%（1/2）	-
**Male/Female**			18/1	17/5	-	*P* = 0.257
**Age (years)**	< 45		14	14	50%（14/28）	*P* = 0.491
	≥ 45		5	8	38.46%（5/13）	
**Site**	lateral ventricle&basal ganglia		12	12	50%（12/24）	*P* = 0.816
	Supra& infratentorial		6	8	42.86%（6/14）	
	Spinal canal		1	2	33.33%（1/3）	
**Blood CD4+T cell count(cells/μl)**			75.11±160.23	260.77±309.44	-	*P* = 0.028*
**Blood HIV viral load(copies/ml)**			251035.11±514865.75	77575.18±134710.78	-	*P* = 0.136
**Blood EBV nucleic acid (copies/ml)**			93785.50±201020.91	18775.56±37790.11	-	*P* = 0.154
**LDH (U/L)**			340.532±264.0347	298.93±167.87	-	*P* = 0.566
**CSF protein (mg/dl)**			129.40±88.16	115.838±94.80	-	*P* = 0.678

**CSF sugar (mmol/l)**		2.93±0.75	3.13±1.42	-	*P* = 0.629
**CSF chloride (mmol/l)**		114.76±31.15	121.247.55±	-	*P* = 0.430
**necrotic areas**	≤ 25%	7	15	31.82%（7/22）	*P* = 0.011*
	26-50%	2	3	40%（2/5）	
	≥ 50%	10	4	71.43%（10/14）	
**CD10**	> 30%	2	7	22.22%（2/9）	*P* = 0.140
	≤ 30%	17	15	53.13%（17/32）	
**BCL-6**	Positive	1	7	1/8	*P* = 0.050*
	negtive	18	15	18/33	
**MUM-1**	Positive	19	19	19/38	*P* = 0.235
	negtive	0	3	0/3	
**BCL-2**	≥ 50%	11	15	11/27	*P* = 0.193
	＜ 50%	8	7	8/15	
**C-MYC**	≥ 40%	10	4	10/14	*P* = 0.131
	＜ 40%	12	15	12/27	
**P53**	≤ 25%	4	2	4/6	*P* = 0.663
	26-50%	4	4	4/8	

	＞ 50%	13	14	13/27	
Ki67	21-50%	3	2	3/5	*P* = 0.519
	＞ 50%	16	20	16/36	
DELs	yes	3	4	3/7	*P* = 0.591
	no	15	17	15/32	
EBER	positive	16	14	16/30	*P* = 0.173
	negtive	3	8	3/11	
	TPS = 0	9	13	9/22	
	TPS = 1-20%	5	5	5/10	*P* = 0.447
	TPS = 21-50%	3	1	3/4	
PD1	TPS>50%	2	3	2/5	
	CPS = 0-19	7	8	7/15	
	CPS = 20-49	4	7	4/11	*P* = 0.615
	CPS = 50-100	6	6	6/12	

CPS>100	2	1	2/3

NOTE. Continuous variables are presented as mean ± SD or median. GCB = germinal centre B-cell like diffuse large B-cell lymphoma, ABC = activated B-cell like diffuse large B-cell lymphoma, BL = Burkitt lymphoma, HIV = human immunodeficiency virus, EBV = Epstein-Barr virus, LDH = dehydrogenase, CSF = cerebrospinal fluid, TPS = tumor cell proportion Score, CPS = combined positive score, DEL = double expressor lymphoma, EBER = Epstein-Barr encoding region. Student's t-test，chi-square test and rank correlation were used.

* $p < 0.05$.

Supplementary Table S3. PDL1 protein and gene in tumors.

Reference	Tumor	PDL1 protein expression (positive rate)	Clinically relevant pathological indicators	*PDL1* gene (gain/amplification rate)	Clinically relevant pathological indicators	Correlation between *PDL1* protein and gene	Impact on prognosis
Koh (2019), Inoue (2016) ,Goldmann (2021)	non-small-cell lung cancer	40-48.3% in adenocarcinoma and 42.3%-49% in squamous-cell carcinoma	smoking-related tumors and hypoxia-related protein, higher risk for lymph node metastasis at diagnosis	3.1-4.5% amplification	high immune infiltrates and EGFR expression	Yes	both PDL1 amplification and level of protein expression were predictors of poor survival (Inoue)/None (Koh, Goldmann)
Wang (2016)	gastric cancer	49.5%	age, histology, tumor size, depth of invasion, lymph node metastasis, lymphatic vessel invasion, venous invasion, disease stage	-	-	-	PDL1 expression was related to a poor prognosis
Billon (2019)	adrenocortical carcinoma (ACC)	49.3% (high express)	younger patients, higher Ki67 index	13.9%	-	-	PDL1 expression is associated with longer postoperative survival
Zhang (2024)	small cell carcinoma	28.6% (CPS)	smaller tumor size	-	-	-	PDL1 expression prolonged

	of the esophagus		and lower T stage				relapse-free survival (RFS)
Cottrell (2018)	inflammatory myofibroblastic tumor	69%	bigger tumor size	-	-	-	
Rotman (2020)	cervical cancer	41%	-	2% amplification	-	None	-
Pérottet (2020)	advanced melanomas	8.33%	-	13.8% amplification and 22.2% gain	-	None	-
Straub (2016)	squamous cell carcinoma of the oral cavity	45%	higher risk for lymph node metastasis	19%	-	Yes	PDL1 expression was related to a poor prognosis.
Berghoff (2013), Chapuy (2016)	primary central nervous system lymphoma (PCNSL)	10%	-	67% (EBV-)	-	-	PDL1 protein expression were associated with poorer OS, but not an independent predictor of OS
Georgiou (2016), Manso (2021), Kiyasu (2015), Cheng (2018), Xu-Monette (2018), Wang (2019)	diffuse large B-cell lymphoma (DLBCL)	26.4% to 75% Positive	non-GCB subtype and EBV positivity	3-3.5% amplification and 6-19% gain	younger age, non-GCB subtype of DLBCL		PDL1 overexpression in DLBCL is validated to be an independent predictor of poor prognosis, especially in ABC-DLBCL (Kiyasu, Cheng)/Green proved that tumor PDL1 expression has been associated with either poor prognosis or no significant prognostic effect in DBLCL (Xu-Monette)/9p24.1

							amplification had a trend of better event-free survival in DLBCL (Wang)
Xu-Monette (2016)	classical Hodgkin's lymphoma (cHL)	70-100%	-	97% copy number alterations	-	Yes	*PDL1* gene amplification and PDL1 expression associated with poorer prognosis
Twa (2014), Camus (2023), Georgiou (2016)	primary mediastinal B cell lymphoma (PMBCL)	56.2%	-	29% amplifications	-	Yes	*PDL1* gain was a unfavorable prognostic factor
Manso (2021)	peripheral T cell lymphoma (PTCL)	12.5%	-	None	-	None	-
Gerbe (2019)	ALK-negative anaplastic large cell lymphoma (ALCL)	46.2%-63.6%	p-STAT3 positive expression	21.1% and 8.3% of systemic and cutaneous ALK-negative ALCL	-	None	None
Ramsay (2012), Gamaleldin (2021), Menter (2016), Zhang (2016)	chronic lymphocytic leukemia (CLL)	-	significantly higher in EBV(+) modified staging in CLL	-	-	-	PDL1 expression was associated with poor prognosis (Ramsay, Gamaleldin)/PDL1 expression had no prognostic significance (Menterand, Zhang)
Loharamtaweet hong (2019)	HIV-positive cervical cancer	56.3%	HIV status, ART	6.5%	ART	Yes	*PDL1* gains and PD-L1 overexpression indicated poor prognosis

NOTE. HIV = human immunodeficiency virus, EBV = Epstein-Barr virus, CPS=combined positive score, GCB = germinal centre B-cell like diffuse large B-cell lymphoma, ART =

antiretroviral therapy.
